# Decision‐Making Balance in Co‐Produced Priority Setting for a Parent Carer Empowerment Intervention

**DOI:** 10.1111/hex.70952

**Published:** 2026-10-27

**Authors:** Nezihe Köker Özer, Ercan Yılmaz, Didem Tatar

**Affiliations:** ^1^ Department of Pediatrics, Faculty of Medicine Inonu University Malatya Türkiye; ^2^ Pediatric Allergy and Immunology Department, Faculty of Medicine Inonu University Malatya Türkiye

Dear Editor,

We read with great interest the study by Reeder et al., which co‐produced a Theory of Change (ToC) to guide a new intervention to empower parent carers of children with neurodisability [1]. The reporting of the process is unusually transparent, and the resulting intervention is grounded in lived experience as well as in theory and evidence. We would like to draw attention to one step in that process, because it carries more weight than any other and because the study's own subject matter makes it worth examining.

The working group comprised nine members: four parent carers, three allied health professional service providers, one service manager and one representative from the commissioning service. In the priority‐setting activity, each member cast two votes, with a rationale, and from that vote the group selected two of thirteen medium‐term outcomes. Those two outcomes, effective two‐way information sharing and trusting relationships between service providers and parent carers, then determined the focus of the intervention and, through the subsequent behavioural analysis, the seven service provider behaviours it targets.

This is the decision that narrows the whole ToC. It is also the decision in which parent carers held 8 of the 18 votes, and service providers, the manager and the commissioner held 10.

The observation matters here more than it would in most co‐production studies, because of what the group itself identified. Among the causal pathways the group mapped was one named around addressing the power imbalance, with a long‐term outcome calling for a change in professional culture related to the privileging of medical knowledge. The asymmetry the group named as a target was also present, in a procedural form, in the room that chose the target.

We are not suggesting the outcome of the vote was distorted. The two prioritised outcomes are directed at service provider behaviour rather than at parent carers, which is consistent with the conclusion the authors drew from their earlier qualitative work, and a differently balanced group might well have chosen the same two. The difficulty is that readers cannot tell. The paper reports the voting rule and the result, but not how votes were distributed across participant groups, or whether the two chosen outcomes drew support from parent carers, from professionals, or from both.

This gap sits awkwardly beside the study's own standard. The authors argue, rightly, that a ToC should be documented transparently so that the underlying change mechanisms can be understood and the work replicated or adapted elsewhere [1, 2]. Prioritisation is the point at which most of the possible interventions are discarded, and it is the step for which the least procedural detail is given. A reader in another service who wished to adapt this ToC would know what was chosen, but not on whose reading of the evidence.

We would also note that the mitigation the authors offer for their small sample, ongoing consultation with the expert research team and the broader involvement group before, during and after the workshops, addresses breadth of view rather than balance of decision‐making. Adding further professional and research perspectives around a group already weighted toward professionals does not change where the votes sat.

For readers, the practical message is that in co‐production work whose target is an asymmetry of power, the decision procedure is not merely method: it is part of the evidence for the claim being made. Three things are cheap to do and would let readers judge it. Report vote distribution by stakeholder group alongside the result. State whether the group's composition was a deliberate design choice, and on what reasoning, since a group balanced for service representation and a group balanced for experiential authority are different instruments. Where a study sets out to shift a professional‐lay imbalance, consider running priority setting in separate strands and comparing them before merging, so that divergence becomes visible rather than being absorbed into a single tally [3, 4].

For the work that follows, the authors' own programme offers a natural check. Stage 3 will test the feasibility of the intervention in the clinical setting. That stage could ask parent carers, including those outside the original working group, whether the two prioritised outcomes are the ones they would have chosen, and report the answer. If they are, the ToC gains something it cannot gain from the workshop alone; if they are not, that is more useful still, and would be worth knowing before the intervention is adapted for other services.

We thank the authors for a carefully documented study, and for reporting their process in enough detail to make this kind of discussion possible.

## Author Contributions

**Nezihe Koker Ozer:** supervision, conceptualisation, writing – original draft. **Ercan Yılmaz:** supervision, conceptualisation, writing – original draft. **Didem Tatar:** supervision, conceptualisation, writing – original draft.

## Policy on Using ChatGPT and Similar AI Tools

The authors used GeminiPro AI for language editing.

## Funding

The authors have nothing to report.

## Ethics Statement

Ethics approval and informed consent were not required for this study, as it did not involve human participants, clinical patient data, or animal subjects.

## Consent

The authors have nothing to report.

## Conflicts of Interest

The authors declare no conflicts of interest.

## Data Availability

Data sharing is not applicable.
